# It Is Movement All the Way Down: Broken Rhythms and Embodied Selfhood in Depersonalization

**DOI:** 10.3390/bs15081090

**Published:** 2025-08-12

**Authors:** Veronika Alekseeva, Anna Ciaunica

**Affiliations:** 1 Department of Psychology and Cognitive Sciences, University of Trento, 38068 Rovereto, Italy; 2 INESC-ID/CEECIND Fciencias.ID, GAIPS, Instituto Superior Tecnico, University of Lisbon, Rua Alves Redol, 9, 1000-029/1049-001 Lisbon, Portugal; 3 Institute of Cognitive Neuroscience, University College London, London WC1N 3AR, UK

**Keywords:** self, somatosensory attenuation, active inference, depersonalisation

## Abstract

From the moment we are born, and even before, in the womb, and until our last breath, our bodies move all the time. Adaptive behaviors necessarily depend not only on the successful integration of multisensory bodily signals but also on how we move our bodies in the world. This paper considers the notion of embodied selfhood through the perspective of dynamic and rhymical coupling between bodily movements and bodily actions. We propose a new theoretical framework suggesting that the dynamic coupling between bodily movements and bodily actions in the world are fundamental in constructing and maintaining a coherent sense of self. To support this idea, we use the Predictive Processing (PP) and Active Inference frameworks as our background theoretical canvas. Specifically, we will focus on the phenomenon of somatosensory attenuation in relation to dynamic selfhood and argue that rhythmic bodily signals such as heartbeats, breathing, and walking patterns are predictable and, thus, can be smoothly attenuated, i.e., processed in the background. We illustrate this hypothesis by discussing the case of Depersonalization Disorder as a failure to self-attenuate self-related information processing, leading to feelings of unreality and self ‘loss’. We conclude with potential implications of our hypothesis for therapy.

## 1. Introduction

From the moment we are born, and even before, in the womb, and until our last breath, our bodies move all the time, both intentionally and unintentionally. Throughout our life, we are constantly immersed in a cascade of sensory signals (smells, sounds, images, etc.) arising from both inside and outside our moving bodies. The fine-grained and flexible orchestration of these dynamic sensory inputs ‘on the fly’ into a coherent sense of self is key for successful navigation in an unpredictable and noisy world. Much theoretical and empirical work has demonstrated that bodily sensations and perceptions scaffold the familiar subjective experience of being an embodied I, present in the here and now (Apps & Tsakiris, 2014; Seth, 2013; Limanowski & Blankenburg, 2013; Ciaunica et al., 2021; see Qin et al., 2020 for a review).

The predominant focus on brain-centered theories of human selfhood has recently shifted towards more embodied views (Gallagher, 2000; Zahavi, 2005; Legrand, 2006; Thompson, 2007). Seminally developed by F. J. Varela et al. (1991) and inspired by the pioneering work of Merleau-Ponty (1945/1962), the key idea is that self-awareness is a dynamic phenomenon emerging first and foremost as basic sensory processing within living organisms such as human bodies situated in a wider environment (de Jaegher & Di Paolo, 2007; Ciaunica et al., 2021; Bennett et al., 2024). This body-centered refocus is supported by the substantial empirical evidence outlining the key intertwined role of dynamic sensorimotor processing and neural processing in constituting human self-experiences (F. J. Varela et al., 1991; see Qin et al., 2020, for a review). The body also serves as the primary interface between sensory perception and social cognition, shaping how we interpret both physical and social realities through bodily cues like posture and gait (Dapor et al., 2023). The replacement of the solitary homunculus in the brain trying to “make sense” of the hidden outer worldly and bodily inputs is particularly welcome in light of growing evidence suggesting a close relationship between brain and body signal processing related to the self (Damasio, 2000; Park & Tallon-Baudry, 2014).

A robust body of work demonstrated that the mechanisms underlying the interplay between exteroceptive, interoceptive, and proprioceptive self-related sensory signals are key to understanding the sense of self and its alterations (Tsakiris, 2017; Park & Blanke, 2019; Azzalini et al., 2019; Koreki et al., 2021). Multisensory integration refers to the capacity to accurately integrate information from both (a) exteroceptive systems, which refer to the classical sensory modalities for perceiving one’s body and the external environment (e.g., vision and audition), and (b) interoceptive senses, i.e., the afferent information arising from within the body (e.g., temperature, itch, pain, cardiac signals) (Craig, 2002; Seth, 2013). In addition, (c) proprioceptive inputs provide sensory information (i.e., from muscles, joints, and vestibular organs) about body position and motion in space. Recent research in healthy adults has revealed interactions between the exteroceptive, interoceptive, and proprioceptive processing of self-related information (Ainley et al., 2012; Chancel & Ehrsson, 2023).

The key idea is that rather than treating these internal and external sensory streams as separate contributors to selfhood, it is crucial to highlight their continuous interplay within a unified system of bodily self-consciousness (Tsakiris, 2017; Park & Tallon-Baudry, 2014; Park & Blanke, 2019). Hence, a comprehensive understanding of bodily self-consciousness must incorporate the dynamic contributions of both internal and external signals. More radically, it has been proposed that interoception may be defined as the integrative interpretation of both internal and external stimuli within a cognitive and emotional context and not just the internal ones (Quigley et al., 2021; Ainley et al., 2016).

Importantly, our bodily self is a flexible, fluid, and dynamic system, continuously updated in the face of constant interactions with an everchanging environment (Tajadura-Jiménez et al., 2015; Stanton & Spence, 2020). A significant body of philosophical, neuroscientific, and psychological research has indeed demonstrated that body representations are malleable and continuously updated by multisensory information received during bodily interactions with the environment (Legrand, 2006; Tsakiris, 2017; Tajadura-Jiménez et al., 2012, 2015; Azañón et al., 2016; Brianza et al., 2019; Stanton & Spence, 2020).

For example, studies using the rubber hand illusion (RHI) (Botvinick & Cohen, 1998) demonstrated how synchronous multisensory inputs can alter the sense of body ownership, creating a vivid sense that a rubber hand is part of one’s body (Ehrsson et al., 2005; Tsakiris et al., 2010; Rohde et al., 2011; Suzuki et al., 2013). By contrast, disruptions in this integration, such as asynchronous inputs, weaken the sense of ownership. In a similar vein, the ‘cardiac rubber hand illusion’ (Suzuki et al., 2013) demonstrated that the strength of body ownership can be enhanced by synchronizing visual feedback with interoceptive (cardiac) signals in time with an individual’s heartbeat. Another study used cardio-visual stimulation in the Enfacement Illusion (Sel et al., 2017), showing that synchronizing a pulsing shade with a participant’s heartbeat increased self-identification with a morphed face and enhanced neural responses to heartbeats, as measured by Heartbeat Evoked Potentials (HEPs).

Similarly, the “embreathment” illusion (Monti et al., 2020) demonstrated that mapping real respiratory patterns onto a virtual body significantly alters corporeal awareness. This study showed that breathing is equally as crucial as visual appearance for body ownership and even more important for body agency. Also, Kosuge et al. (2023) showed that respiratory rhythms recalibrate body ownership, where congruency between respiratory signals and external stimuli facilitated ownership transfer over the fake hand. Interestingly, Adler et al. (2016) demonstrated that synchronous visuo-respiratory feedback increases self-location and breathing agency toward a virtual body, even when participants’ breathing was machine-assisted, highlighting respiratory signals’ interoceptive contributions to bodily self-consciousness. Importantly, both active and passive breathing conditions modulate self-identification with a virtual body equally during visuo-respiratory synchronization (Betka et al., 2020).

Previous work outlined the importance of rhythmic physiological activity shaping neural development in utero and beyond (Bastos et al., 2020; Wolpert & Tallon-Baudry, 2021; Corcoran et al., 2023/2025). Indeed, one important yet overlooked aspect is that the bodily self is intrinsically related to the processing of self-related signals in relation not only to the body (F. J. Varela et al., 1991) but, importantly, in relation to the body in movement. Human bodies are indeed highly dynamic systems, constantly moving both inside (e.g., heartbeats and breathing) and in the outside world (e.g., footsteps and walking) to secure survival.

In this theoretical paper, we propose to take a fresh look at the notion of embodied selfhood by using, as an entrance point, the dynamic coupling between bodily movements and bodily actions. Specifically, we propose to conceptually distinguish between (a) bodily movements (e.g., cardiac and respiratory signals). For example, my heart beats all the time, even when I am asleep or I am in the womb, that is, without explicit conscious awareness of my movements. Also, when I breathe, my chest is moving following the inspiration/expiration pace. Bodily movements are thus fundamental and pervasive aspects of living organisms such as humans. By contrast, (b) bodily actions refer to the voluntary, agentic, and intentional movements that one may take in the world (Haggard, 2017). For example, I can actively and intentionally reach for a glass of water or walk to the grocery store or into the woods to obtain food. Importantly, survival depends on the successful coordination of both bodily movements and bodily actions, in addition to the flexible multisensory integration of bodily signals from inside and outside the body, as demonstrated through the RHI. Hence, we need to expand our picture of embodied selfhood to encompass this intrinsic relationality, coupling movements and actions as a necessary fundamental component of the self.

This paper proposes a new theoretical framework suggesting that the dynamic coupling of bodily rhythms—inner movements (such as the cardiac cycle) and outer actions (such as the walking cycle)—is fundamental in constructing and maintaining a coherent sense of self acting in the world. To support this idea, we use the Predictive Processing (PP) (Friston, 2005; Clark, 2013; Hohwy, 2013) framework as our background theoretical canvas. We build upon the observation that, necessarily, living systems are moving systems that need to effectively couple rhythmical dynamical sensory signals from inside the body (i.e., cardiac and respiratory signals) with rhythmical actions in the world (i.e., walking). The key idea is that it is the process of coupling bodily movements and bodily actions which facilitates the seamless integration of interoceptive and exteroceptive cues, and not just the multisensory integration of inner and outer bodily signals. Indeed, bodily movements and bodily actions in the world ground the body within its volatile environment by keeping a stable sense of agency and ownership within a precarious homeostatic balance maintained via energetic exchanges with the environment (Damasio, 2000; Maturana & Varela, 1980). When this fragile balance is disrupted, instead of fixing the self alone, or the individual per se, one needs to address the relationship between the individuals’ bodily movements and actions in the physical and social world.

This paper is set up as follows:

In Section 2, we emphasize the dynamic aspect of multisensory integration—specifically, the importance of coupling bodily actions and bodily movements in constituting our sense of self. In Section 3, we use the PP Bayesian framework to show that human bodies are indeed biological self-organizing living systems “on the move” and under constant pressure to resist entropic dissipation, and as such, they live under drastic time constraints and limited energetic resources (Ciaunica, 2025). Because of these inherent limitations, human organisms need to learn very early on what information to process or attend, and what information to dis-attend or attenuate. Hence, the process of “ignoring” or attenuating sensory information is as key as the process of acquiring information for a living system such as the human body (Ciaunica et al., 2021; Ciaunica, 2025). Specifically, we will focus on the phenomenon of somatosensory attenuation in relation to dynamic selfhood and argue that rhythmic bodily signals such as heartbeats, breathing, and walking patterns are predictable and, thus, can be smoothly attenuated, i.e., processed in the background. We illustrate this hypothesis by discussing the case of Depersonalization Disorder (Sierra & Berrios, 1997) in Section 4 and Section 5 as a failure to self-attenuate self-related information processing, leading to feelings of unreality and self ‘loss’ (Ciaunica et al., 2022; Ciaunica, 2024). We conclude in Section 6 by discussing the potential implications of this shift in focus for potential therapy, based on dynamic rhythmical embodied interventions.

## 2. Living on Rhythms: Coupling Bodily Movements and Bodily Actions

Life is movement and movement is life (Thompson, 2007). Living organisms like human bodies constantly pulsate to rhythms. The study of sensory visceral signals originating from inside the body such as cardiac (Craig, 2002; Garfinkel et al., 2015), gastric, and respiratory (Engelen et al., 2024) rhythmical signals has been recently placed at the very core of human bodily self-consciousness (Park & Tallon-Baudry, 2014; see also Carpenter et al., 2025). A significant body of theory and empirical work illustrates that the sense of a stable and continuous sense of self emerges more robustly from neural representations of rhythmically patterned visceral signals than from less predictable and transient exteroceptive inputs. For example, Park and Blanke (2019) note that signals tied to the hands, for instance, vary widely depending on our interactions with the environment, producing relatively brief and irregular neural responses. In contrast, visceral inputs are continuously conveyed to the brain in cyclical patterns that help maintain self-regulatory processes such as homeostasis (Damasio, 2000) within a regulated physiological range.

Indeed, there is growing evidence supporting the role of rhythmical visceral signals, such as cardiac phases, in modulating sensory processing. Studies indicate that sensory precision for exteroceptive inputs, such as visual and tactile stimuli, fluctuates across the cardiac cycle. For example, Azevedo et al. (2017) found increased race-driven misidentification of weapons during systole, highlighting how interoceptive signals modulate sensory processing in alignment with baroreceptor activity. Similarly, Galvez-Pol et al. (2020, 2022) provided evidence that visual and tactile precision is modulated by cardiac phases, with diastole favoring heightened sensitivity to external stimuli. While we actively spend more time sensing during systolic periods, Saltafossi et al. (2023) further corroborated these findings, showing phase-dependent variations in multisensory integration. This attenuation of exteroceptive signals during systole is hypothesized to arise from interoceptive signals competing with exteroceptive inputs for attentional resources (Ren et al., 2024).

This modulation is believed to optimize the sampling of environmental stimuli by aligning sensory attenuation with periods of increased baroreceptor activity during systole. Baroreceptor signals, triggered by the pulsatile ejection of blood from the heart, prioritize internal homeostasis and modulate sensory processing to reduce sensitivity to external stimuli. This process prevents overstimulation from redundant sensory inputs and enhances the capacity to detect novel or salient environmental changes during diastole, when sensory precision is higher. As we will see shortly in the next section, this phase-dependent fluctuation in sensory processing aligns with the PP account, allowing for the efficient allocation of attentional and neural resources (Critchley & Garfinkel, 2018; Al et al., 2020; Allen et al., 2022).

An examination of the dynamic coupling between cardiac and respiratory signals and locomotor signals is key to our argumentation here. Indeed, our biological survival depends on the ability to rhythmically act in the world (i.e., walking and running) to secure nutrient resources and social support, and these actions are critically linked to the rhythmical movements of the cardiac and respiratory processes.

Also, a recent study suggested that the relative timing of core body temperature and melatonin rhythms may be key circadian features linked to depressive symptoms (Carpenter et al., 2025).

Previous work showed that real-time modulation of the sounds (modulation of sound frequency spectrum) produced by one’s own footsteps while walking can modify individuals’ body perception, emotional states, and even their gait patterns, which constitute an implicit behavioral indication of changes in body representations (Tajadura-Jiménez et al., 2015, 2017; Gomez-Andres et al., 2020; see also Ley-Flores et al., 2021, 2022).

Supporting this hypothesis, studies investigating full-body movements during locomotion offer additional insights into the complexity of bodily self-consciousness and its relation to everyday bodily actions such as walking. For example, cardiac–locomotor synchronization (CLS) is a well-documented physiological phenomenon wherein heart rate becomes coupled with locomotor rhythms during movement. Studies have shown that this synchronization is not just a byproduct of mechanical movement but an emergent property of the interaction between the cardiac and locomotor systems. Interestingly, Kirby et al. (1989) demonstrated that during locomotion, the heart rate can become entrained with the stride rate (the number of steps per minute), particularly at moderate-to-high exercise intensities. This coupling is hypothesized to optimize blood flow to muscles by aligning peak arterial pressure with the phase of muscle relaxation, reducing cardiac load and enhancing endurance performance.

Also, de Carvalho et al. (2021) expanded on this concept by investigating spontaneous CLS in long-distance runners. They found that at higher running speeds, the heart rate and step frequency became naturally synchronized, suggesting that CLS emerges in response to the physiological demands of endurance exercise. Their findings support the idea that CLS could contribute to improved metabolic efficiency and performance by optimizing oxygen delivery to working muscles. Rosato et al. (2024) further explored spontaneous cardiac–locomotor coupling in everyday movement, showing that this synchronization is not limited to high-intensity exercise but also occurs during natural locomotor activities. Their study underscores the fundamental role of CLS in maintaining physiological stability and optimizing energy distribution across different levels of physical exertion.

Further evidence from Kannape et al. (2010) demonstrated that individuals consciously monitor their body’s spatial location while walking with notably low accuracy, and this can be systematically influenced by experimental conditions. Extending these findings, Kannape and Blanke (2012) showed that gait movements—being cyclic, less overtly goal-directed, and more automatic—differ fundamentally from the more discrete, hand-centered actions typically studied in agency research. This slower, more regular input may align more closely with the temporal properties of visceral signals, enhancing the efficiency of their multisensory integration and reinforcing the stable experiential presence of the self. Also, Kaneno et al. (2024) reported that in static conditions, the impact of interoceptive sensitivity on body ownership was inconsistent and could not be reliably replicated. However, in dynamic contexts, such as the moving RHI, voluntary movements played a critical role in enhancing the sense of agency and ownership.

Taken together, the theoretical and empirical work reviewed so far suggests that our sense of self crucially depends not only on the multisensory integration of sensory signals from inside and outside the body but also on the dynamic coupling between bodily movements and bodily actions. The adaptive relevance of this coupling makes sense from an evolutionary perspective in a world where living systems need to constantly negotiate resources with the environment to keep one’s homeostatic balance within the optimal range for survival (Damasio, 2000). To put it provocatively, it is not enough to statically perceive a red tomato; it is also important to coordinate bodily movements and bodily actions to reach the tomato in the real world and eat it. Without these constant engagements with the world designed to provide the organism with the means to intake energy to resist decay, the bodily self and human organism would simply disintegrate and die.

## 3. Sensing and Attenuating the Self Through Movement

The importance of the active and interactive component of embodied selfhood has been recently linked to the influential Predictive Processing (PP) framework, stipulating that incoming sensory signals need to be adaptively processed and integrated in order to keep our bodies’ homeostatic balance within the required limits for survival purposes (Friston, 2005). Thus, to cope with uncertainty in a potentially threatening environment, the human brain needs to reliably predict or infer the ‘hidden’ causes of incoming sensory information (Hohwy, 2013; Clark, 2013). This idea builds upon a long tradition going back to the groundbreaking work of Helmholtz (1866/1962) and Conant and Ross Ashby (1970).

The basic idea is that incoming sensory input is processed by comparing it to (learned or innate) prior beliefs or expectations about what constitutes typical sensory input, given ‘the kind of creature that I am’ (Ramstead et al., 2019). The brain thus generates probabilistic, Bayesian, and self- and world models that are constructed moment by moment at various levels of hierarchical processing. When a prediction does not match ongoing sensory input, a ‘prediction error’ (PE) results, which may have the effect of updating the initial prediction. The dynamic, fine-grained, and flexible orchestration of sensory inputs and inferential processes aims primarily at guiding our perceptions and daily embodied actions in the world. Through the lens of PP, it has been argued that sensory systems, including interoception, are shaped by evolutionary pressures to optimize energy efficiency (Ainley et al., 2016). Indeed, interoceptive signals, such as those indicating energetic deficits, must integrate with external sensory inputs like vision and smell to coordinate adaptive behaviors like foraging.

**Figure 1:**
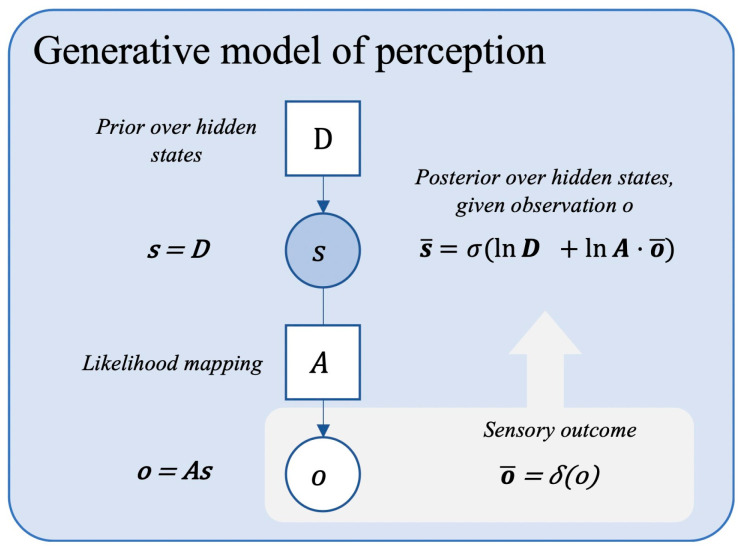
A simple generative model. Quantities in open circles have to be inferred and are updated in inference (within a given trial), while parameters are updated on the longer timescale of learning (between trials). Adapted with permission from Sandved-Smith et al. (2021); based on templates from Hesp et al. (2021).

Importantly however—and this is our key point here—adaptive behaviors necessarily depend upon the successful integration of multisensory signals coupled with bodily movements (i.e., heartbeats, respirations, etc.) and bodily actions. Survival cannot be achieved without bodily actions in the world, and this aspect is crucially captured by the Predictive Processing framework. According to the Predictive Processing framework, the brain actively infers what is causing its incoming sensory inputs based on probabilistic beliefs about the causal structure of the process (i.e., the causal factors) that generated its sensory signals or observations (Parr & Friston, 2017). These beliefs are harnessed within a so-called generative model (see Figure 1). The brain takes two key routes to optimize the evidence for its generative models: (a) by changing (updating) the model to better account for sensory samples (perceptual inference); (b) selectively sampling sensations that can be accounted for by the model (Active Inference) (Adams et al., 2013; Friston et al., 2017).

The difference between the sensory data that is expected under the generative model and the actual data observed is quantified by a construct called variational free energy (Ramstead et al., 2019). Heuristically, this free energy can be interpreted as a measure or approximation of how much (negative model) evidence is provided by our sensory data for the hypotheses harnessed in the generative model (Friston, 2005). This affords the theory an inferential or Bayesian aspect: self-evidencing is formally equivalent to tracking and minimizing the variational free energy that is generated by discrepancies between predicted and sensed data. In short, self-organizing systems need to constantly prove to themselves that they exist in a dynamic, constantly changing, and noisy world, by fulfilling their beliefs about action: they are their own existence proof (Friston, 2018).

From this point of view, we experience ourselves as being endowed with a persisting self because we reliably infer that the existence of such a self is the most probable cause of our sensory observations. As Ramstead and colleagues note, the fact that my experiences seem to have a zero point or origin that I carry along with me as I move my body is best explained by postulating that there exists a causal factor. This causal factor is an I or self, which is attached to a body and that is distinct from other selves (Ramstead et al., 2019). The key idea here is that embodied agents coordinate and maintain probabilistic models of their worlds or ecological niches, which they use to generate situationally appropriate forms of behavior (Constant et al., 2018; Ramstead et al., 2019). A very simple generative model, which is capable of rudimentary, moment-to-moment perceptual inference and confidence estimation, is depicted in Figure 1.

Here, observations (denoted as o) comprise all the sensory data that is available to the agent. Hidden states (denoted as s) are hypotheses about what might have caused the observations and essentially capture the ontology or set of representations that the agent is using to parse its sensory stream. The parameters modulate the inference process and harness prior beliefs about the process that generates sensory data. The likelihood mapping (denoted A) harnesses beliefs about the relation between states and observations: it is a matrix that lists the probability of some observation, given some state, i.e., the probability of sensing this, given that that is the case. The model contains a baseline prior over hidden states (denoted as D), which specifies beliefs about the baseline probability of states, independent of observations. The framework is broadly Bayesian in that it specifies how to optimally combine prior beliefs about some process with current sensory evidence (which is what Bayes’ rule formalizes). Posterior beliefs are hypotheses concerned with the causes of sensory input that are derived by optimally combining prior beliefs about the self and world and current sensory evidence.

Crucially, it has been argued that in updating the self and world models, much depends on the ‘precision’ of the (i) prior prediction and (ii) the prediction error (PE) caused by the incoming sensation (2018). Prior beliefs and sensory data are represented as probability distributions with mean values (expectations) and precision (inverse variance). (1) If a prediction error emerges from the contrast of precise sensory data and relatively imprecise prior beliefs, the mean of the posterior will be closer to the mean of the sensory data. (2) If the imprecision comes from sensory information, posterior beliefs will be much closer to prior beliefs (see Yon & Frith, 2021).

Importantly, this multi-layered modeling system needs to ‘decide on the fly’ whether the weight of the balance—the ’gain’ of the updating process—goes to the (a) sensory evidence coming from different sensory modality or (b) to the prior beliefs (or expectations) that the model puts forward. Switching the influence from prior beliefs to sensory evidence (i.e., prediction errors) or vice versa is implemented via the fine-grained process of “precision weighting”. Indeed, the system is also trying to predict the precision of PEs: we have thus first-order representations of the second-order precision of the hierarchical architecture of the prediction errors. PEs that are expected to convey precise, reliable, high-quality information are afforded greater precision or weight, such as that they have a greater influence on perception. This equates to “attending to the right sort of newsworthy information and attenuating imprecise or ‘fake’ news. (…) It is this mechanistic bridge between a fundamental computational imperative to properly balance sensory evidence against (hierarchical) previous expectations and plausible neurobiological mechanisms that links psychopathology and pathophysiology.” (Friston, 2018, p. 642).

In short, precision optimization is a mechanism that allocates ‘weight’ or ‘gain’ either to sensory input or PEs higher in the hierarchy. Given that precision-weighted processing works as a kind of ‘searchlight’, it has been argued that this makes it a “promising candidate for the mechanism for attention” (Hohwy, 2020, p. 9, original italics). Indeed, the precision of sensory data and prior beliefs is not fixed: attention is roughly the process of optimizing precision in neural hierarchies, such that attended locations or objects are afforded higher precision (Feldman & Friston, 2010). For example, directing attention to a stimulus can increase its perceived intensity (Liu et al., 2009; Carrasco et al., 2004). Previous work showed that the gain or post-synaptic responsiveness can be altered by several factors, including fast synchronous oscillatory activity and/or neuromodulators such as acetylcholine and dopamine, both of which are implicated in attentional mechanisms (Feldman & Friston, 2010).

It has been recently proposed to significantly broaden the concept of attention in order to take into account inner bodily states (interoception) as well as dynamic brain–body coupling (Allen et al., 2022; Quadt et al., 2018). Crucially for our argumentation here, this process is exactly the opposite of sensory and somatosensory attenuation (Blakemore et al., 1998; Waszak et al., 2012). Somatosensory attenuation—the process of dampening the self-generated sensory signals—plays a crucial role in bodily self-awareness and agency and is critical for distinguishing between self and non-self (Hughes & Waszak, 2011; Horváth, 2015). We typically predict and suppress the sensory consequences of our actions which explains, for instance, why we cannot tickle ourselves (Blakemore et al., 1998).

Neurophysiological studies revealed reduced activity in the primary somatosensory cortex during voluntary movements (Bays et al., 2005; Palmer et al., 2016) and show that the cerebellum, via its functional connectivity with primary and secondary somatosensory areas, actively predicts and attenuates the sensory consequences of self-generated touch (Kilteni et al., 2019). Findings from recent neuroimaging meta-analyses provide further support that sensory attenuation is mediated by a cerebellum-centered action prediction network that suppresses reflexive inputs elicited by self-initiated actions (Gu et al., 2025).

From an Active Inference perspective, sensory attenuation is essential for initiating an action and making the “self-fulfilling prophecy of acting” come true (Brown et al., 2013). To ensure that predicted action outcomes align with actual movements, proprioceptive prediction errors must be attenuated by reducing their precision, preventing contradictory sensory evidence that could disrupt the sense of agency (Adams et al., 2013; Seth & Friston, 2016). In this way, the stability of self-representation is established, differentiating the agentic self from external influence.

During sensory attenuation, attention is indeed withdrawn from the expected consequences of movement, such that movement can occur. Sensory attenuation can thus be regarded as the complement of attention, necessary for generating action: attenuating or augmenting the gain (precision) afforded to self-generated sensory prediction errors. This involves the successful suppression of the prediction error signal that would otherwise drive the system to resolve that prediction error through perception, i.e., by changing the hypothesis, rather than by making the sensation conform to the hypothesis (Friston, 2018; Adams et al., 2013; Brown et al., 2013; Seth & Friston, 2016). Thus, with regard to action, precision weighting entails the attenuation of self-generated sensory evidence.

The key idea here is that obtaining the ‘right’ precision estimates means lowering them to attenuate sensory evidence, and this holds especially when modeling oneself: “The temporary attenuation of the precision of sensory “self-evidence”—which is necessary to entertain an alternative and yet counterfactual, (cf. Seth, 2013) hypothesis about myself—is effectively a form of “self-attenuation” (Limanowski & Friston, 2020, p. 10). One may call this basic default mode of self modeling ‘dis-attending’ or ‘transparent self-attending’. By contrast, attending tout court refers to the process of deliberately attending to a set of states, thereby rendering them ‘opaque’, i.e., available to attending awareness (Limanowski & Friston, 2018, 2020).

Previous work demonstrated that failures of self-attenuation are a hallmark of several psychopathological and altered cognitive states. For example, in schizophrenia, impaired attenuation of self-generated sensory signals results in their misattribution to external agents, leading to perceptual distortions and a compromised sense of agency, manifesting in symptoms such as delusions of control and hallucinations (Shergill et al., 2005; Fletcher & Frith, 2009; Brown et al., 2013; Leptourgos & Corlett, 2020). The same mechanism has been recently proposed as fundamental in explaining atypical bodily self-awareness in other self-disorders such as depersonalization (Ciaunica et al., 2021; see also Section 5 and Section 6 below).

This disruption affects only the positive dimension of schizotypy, with reduced attenuation of self-generated touch not observed in its negative or disorganized dimensions (Asimakidou et al., 2022). Similarly, in functional motor and sensory symptoms (‘hysteria’), aberrantly precise priors override sensory evidence, leading to false percepts or involuntary movements—a failure of attenuation that parallels the mechanisms proposed in psychosis (Edwards et al., 2012). In autism, a breakdown in the oxytocin-mediated modulation of interoceptive and somatosensory precision disrupts attenuation, generating a hypersensitivity to internal states that impairs social engagement and the formation of a coherent self model (Quattrocki & Friston, 2014). Anxiety-related disruptions in motor control, such as fear of falling or performance failures, likewise reflect a failure to attenuate proprioceptive prediction errors. In such cases, excessive attention maladaptively up-weights minor fluctuations in sensory feedback, destabilizing motor predictions and eroding fluency (Harris et al., 2023).

Notably, meditative practices appear to bidirectionally influence self-attenuation depending on their attentional orientation. Excessive attenuation of self-generated signals underlies deep meditative states that aim to blur or dissolve perceived bodily boundaries, giving rise to experiences described as “oceanic boundlessness” or “selfless” states (Ciaunica et al., 2021; Ciaunica, 2024). In such conditions, the organism is stabilized into a near-static posture, allowing it to predict its internal bodily states with such precision that self-related sensory information is processed in the background with minimal conscious awareness. However, these states are typically short-lived, as even minor physiological deviations, like an itch or the urge to shift posture, break the illusion and reintroduce the body into conscious awareness.

By contrast, meditative practices that emphasize sustained attention to bodily sensations, such as “body-scanning,” may have the opposite effect by amplifying self-related sensory precision. For example, Poublan-Couzardot et al. (2025) recently explored this possibility using the force-matching paradigm (Wolpe et al., 2016) to assess somatosensory attenuation in experienced meditators across a three-week meditation retreat. Their hypothesis was that enhanced interoceptive awareness cultivated during meditation would reduce somatosensory attenuation, resulting in more accurate force-matching performance. Indeed, they found that somatosensory attenuation was negatively correlated with mindfulness scores. However, the attenuation effect did not significantly change after the intensive retreat. Interestingly, one interpretation offered by the authors is that they did not consider movement during the task. Indeed, skilled meditators are trained not only to attend to bodily sensations but also to monitor their movements. The increased precision of motor predictions (i.e., stronger motor-related attenuation) during the task may have counteracted the reduced attenuation of tactile sensory input.

## 4. Perceiving the World Through Bodily Rhythms

If we start with the observation that our bodies move constantly, then all perception of the self and world is necessarily performed through the lens of a moving system, rather than as a static screenshot. This observation is particularly important if we note that visual perception has been tacitly adopted as the paradigm case of how humans perceive and relate to the world (Faivre et al., 2017; Ciaunica & Fotopoulou, 2017). Hence, we need to reconsider our model of perceptual experiences and selfhood in order to consider the pervasive background of our bodily movements. This important point has been recently outlined by a number of theorists working on bodily awareness in adults and in early life (Park & Tallon-Baudry, 2014; Ciaunica, 2016; Bastos et al., 2020; Corcoran et al., 2023/2025).

For example, Engelen et al. (2023) discuss three frameworks to explain how bodily rhythms like cardiac, respiratory, and gastric cycles interact with brain activity and sample external inputs. These frameworks are (i) oscillatory synchrony, (ii) predictive coding, and (iii) multisensory integration. Oscillatory synchrony in the brain is thought to coordinate large-scale neural dynamics and communication, originally viewed as emerging from purely neural interactions (F. Varela et al., 2001). Several mechanisms to explain how oscillations guide information flow such as “communication through coherence” (e.g., Fries, 2005, 2015) and “gating by inhibition” (Jensen & Mazaheri, 2010) have been proposed. The “scaffolding hypothesis” expands this idea by proposing that bodily rhythms—mainly respiratory and gastric—can serve as carrier waves, creating windows of excitability across the brain that align with those rhythms (Richter et al., 2017; Rebollo et al., 2018; Tort et al., 2018). These bodily driven oscillations seem to not depend much on specific physiological states, and evidence so far is mixed regarding how consistently different brain regions align to them. This raises questions about how bodily rhythms might functionally couple to brain activity. A key unresolved issue is how the ‘brain selects’ which neuronal populations synchronize under these rhythms, though vascular pulsatility transduced by astrocytes and pyramidal cells (Kim et al., 2016; Marina et al., 2020; see Engelen et al., 2023, for a recent comprehensive discussion) could offer fresh avenues to address this question, especially in the case of disturbances of self-experiences.

For example, recent findings on brain–body dysconnectivity in schizophrenia provide compelling evidence that autonomic nervous system activity plays a role in organizing large-scale cortical function. Specifically, phase–amplitude coupling between heart rate variability (HRV) and electroencephalographic (EEG) oscillations has been identified as a key mechanism through which autonomic cycles influence neural excitability (Sargent et al., 2025). This aligns with the scaffolding hypothesis, suggesting that bodily rhythms, including cardiac and respiratory cycles, create temporal windows of excitability that modulate cortical communication. Notably, individuals with schizophrenia exhibit disrupted HRV-EEG coupling, particularly in the theta and alpha bands, which correlates with deficits in sustained attention (Sargent et al., 2025). These findings point toward a more integrated view of neural communication, where bodily rhythms act not just as passive background noise but as an underlying mechanism that modulates neuronal excitability and functional connectivity. The evidence of disrupted cardiac-to-cortical modulation in schizophrenia further supports the view that oscillatory synchrony is shaped by multisystem interactions rather than purely intracortical mechanisms. This also may explain findings such as the suppression of neural responses to tactile stimuli during systole as competition between tactile and baroreceptor inputs.

Neurophysiological findings demonstrate that cardiac signals modulate auditory regularity processing even in the absence of consciousness (Pelentritou et al., 2025). Crucially, stronger coordination between heartbeats and brain responses to deviant sounds with the synchronized heartbeat sequence predicted better chances of coma recovery. These findings offer empirical support for the idea that interoceptive–exteroceptive coupling underpins perceptual integration and highlight the fundamental role of rhythmic (i.e., cardiac) bodily movements.

As Engelen et al. (2023) note, multisensory integration and the Predictive Processing frameworks provide distinct explanations for the relationship between bodily rhythms and self-perception. Multisensory integration focuses on the convergence of interoceptive and exteroceptive signals, often downplaying the role of rhythmic patterns. While the Predictive Processing approach accounts for bodily rhythms in shaping self-perception, it treats these rhythms as just one aspect among many that contribute to the brain’s predictions about bodily states.

While an extensive discussion is beyond the scope of this paper, it is important to note that the PP framework allows us to consider that bodily rhythms, due to their high predictability (i.e., we expect our heart to beat all the time and to breathe all the time), help align neural oscillations with sensory processing. The advantage of this framework is that it extends beyond short-term predictions. It integrates allostatic regulations (Sterling, 2014) that anticipate bodily needs based on external contexts and links these predictions to emotional feelings and, what we argue, the sense of self. This also includes self-disruptions in conditions such as Depersonalization Disorder (Sierra & Berrios, 1997), where people feel disconnected from their self and body. We turn to this discussion now.

## 5. When the Self Becomes Stuck: Altered Somatosensory Attenuation in Depersonalization

Depersonalization (DP henceforth) is a very common phenomenon that makes people feel detached from their bodily self (Sierra & Berrios, 1997). DP is the third most common psychological symptom reported in the general population (after anxiety and low mood) (Simeon et al., 2003). DP is typically characterized by a distressing feeling of being detached from one’s self, body, and the world: “I look in the mirror and it doesn’t feel like myself I’m looking at. It’s like I’m floating, not actually experiencing the world, and slowly fading away into nothing. It’s like I’m on autopilot in somebody’s else body” (Perkins, 2021, p. 198). DP experiences can be triggered by high stress, severe depression, traumatic life events, or drug use (Simeon et al., 2003). Remarkably, despite its high prevalence and the significant distress and social isolation it triggers, the mechanisms underlying the altered sense of self in DP remain poorly understood.

Alterations in attentional processes at early sensory stages (Guralnik et al., 2007; Schabinger et al., 2018), abnormal interoception-related attentional processes (Gatus et al., 2022), and a disrupted integration between interoceptive and exteroceptive processes (disruption in sensory–motor–affective integration) (Salami et al., 2020; Saini et al., 2022) lie at the heart of DP phenomenology. The experience of a ‘split’ between the self and the body at the basic sensory level—strikingly described as feeling trapped in one’s head (mind) and outside one’s body (Ciaunica et al., 2020)—is one of the most frequently cited symptoms in DP (Sierra & David, 2011). This separation is responsible for the ensuing sense of (a) self-detachment, of looking to oneself from the outside, from the “back of one’s eye sockets”; and (b) unrealness, often and strikingly reported by DP patients as ‘having a pane of glass’ interposed between one’s self, body, and the world (Simeon & Abugel, 2006; Ciaunica et al., 2020).

The prevalence of transient episodes of depersonalization is between 34 and 70% in the general population (Hunter et al., 2004). For example, almost 50% of college students report DP experiences (see Salami et al., 2020 for a recent review). Previous theoretical and empirical work demonstrated disrupted physiological responses in patients with DP, compared to healthy participants (Dewe et al., 2018; Owens et al., 2015; Sierra et al., 2002). DP has also been linked to disrupted activity in neuronal regions underlying somatic processing (Lemche et al., 2013; Medford et al., 2016) and the vestibular system (Jáuregui Renaud, 2015), which is responsible for providing information about the body’s position in space (Ferrè & Haggard, 2016).

The mechanisms underlying the interplay between exteroceptive, interoceptive, and proprioceptive self-related sensory signals are key to understanding the sense of self and its disturbances (Park & Blanke, 2019; Koreki et al., 2021). The empirical evidence on interoceptive processing in DP is unclear. For example, some studies found impaired interoceptive processing (Sedeño et al., 2014), while others found normal interoceptive accuracy in DP (Michal et al., 2014). Moreover, altered patterns of heartbeat-evoked potentials have been reported in depersonalization, providing neurophysiological evidence for impaired cortical representation of bodily signals (Schulz et al., 2015). More recently, Cavicchioli et al. (2024) have shown that dissociative experiences may stem from imbalances in temporal integration and segregation of multisensory stimuli (“hyper-segregation” and “hyper-integration” processes). These findings support the idea that dissociation, an aspect of DP, is closely tied to the abnormal processing of sensory signals.

Evidence suggests that DP experiences may be more tightly linked to disruptions in proprioceptive and vestibular processing than to interoception alone. Vestibular dysfunction or sensory conflicts (e.g., mismatches between vestibular and visual inputs) can induce feelings of detachment and unreality (Jáuregui Renaud, 2015). While objective interoceptive accuracy (e.g., heartbeat detection) remains ‘normal’ in DP patients (Michal et al., 2014), they experience a profound feeling of disembodiment. This apparent paradox suggests that DP symptoms are connected not with the loss of internal bodily sensations, but rather with a breakdown in multisensory integration: failure to coherently integrate interoceptive signals with exteroceptive and proprioceptive inputs to maintain an embodied sense of self.

For example, highly trained healthy runners may experience a state of flow where their sense of bodily sense is enhanced, even though they do not keep track mentally and pay explicit attention to every single movement they do. Our theory predicts that DP experiences will decrease after a running session where the individual needs to successfully coordinate breathing and movement. Another example is movement-based yoga training, whereby breathing and movement coordination may enhance the sense of self in people with DP. A straightforward prediction is that people with depersonalization will have more difficulties in coordinating movement and breathing in yoga sessions. Our theory also predicts that individuals who are better coordinators of breathing/movement tandem will also report lower depersonalization experiences after a yoga session.

Despite robust evidence showing that our bodily self is not fixed but is constantly updated through dynamic sensory feedback, including sound feedback (Tajadura-Jiménez et al., 2012, 2015; Stanton & Spence, 2020), little is known about the underlying mechanisms subserving the relationship between self-detachment, bodily movements, and dynamic sensory feedback in DP. This is surprising because DP experiencers instinctively use proximal interactions with others and the environment as a compensatory means to ‘get back into their bodies’: “When the depersonalisation is very deep, (…) it feels like that constant source of interaction is the only thing that allows me to maintain a connection with the world. I’ll also seek physical contact with whoever I’m with.” (Ciaunica & Charlton, 2018). It is crucial thus to address this question because, as one person with DP strikingly puts it: “a disorder that makes you feel invisible is invisible in society” (Perkins, 2021, p. 193).

Importantly, the focus on coupling bodily movements with bodily actions in relation to altered selfhood in DP can be nicely addressed within the Active Inference framework as a manifestation of altered somatosensory attenuation (Ciaunica et al., 2022). Specifically, the key idea is that in typical individuals, highly predictable self-related signals (i.e., “boring” signals) are down-weighted in precision, allowing the brain to prioritize contextually relevant external inputs (i.e., “interesting” new information about the world). However, in DP, all sensory data—internal and external—is experienced with equal intensity, disrupting the adaptive hierarchy of sensory processing. Consequently, the generative model fails to categorize non-attenuated self-related signals as belonging to the self. Instead, the model’s best guess is that these signals are “external”, blurring the boundaries between the self and the world. Hence, people with DP view themselves as strangers to themselves, as objects seen from the “outside” (Simeon & Abugel, 2006; Perkins, 2021).

Indeed, the process of precision weighting may become ‘stuck’, and the self-regulatory balance will lean systematically towards either bottom-up sensory evidence or top-down prior belief at various hierarchical levels, regardless of the relevant contextual inputs. Such a system will automatically lose the flexibility afforded by an adaptive modulation to the incoming sensory information or PEs typically associated with optimal gain and successful self- and world model updating. Hence, disruptions at the level of precision estimation may lead to the system’s inability to dis-attend or to process its self model transparently (Limanowski & Friston, 2020; Ciaunica et al., 2021).

Failures of somatosensory attenuation and consequent abnormal perceptions and beliefs may lead in turn to self-opacity—the self model becomes ‘opaque’, ‘stands in the way’ and hinders the direct access to one’s self and the world. Ensuing subjective feelings of ‘losing touch’ with one’s self, body, and the world may occur (Simeon & Abugel, 2006; Ciaunica & Charlton, 2018). Indeed, because the precision is inferred, belief updating about precision at higher levels of the hierarchy may disrupt the inherent transparency of self modeling. These alterations may trigger attempts to respond by compensatory hyper-cognitive and hyper-reflexive strategies (Fuchs, 2005). However, these compensatory strategies disconnect individuals from moving bodies and get them “stuck in the head”, that is, in the mental realm, to the detriment of actively engaging with the world via embodied interactions.

## 6. Broken Rhythms in the Self: Implications for Therapy

The world is a highly unpredictable place with one major constant or attractor: the self. High predictability of self-related sensory signals anchors the healthy sense of self in effective coupling between bodily movements and bodily actions in the world. Like all living self-organizing biological systems, human bodies need to engage in energy and resource exchanges with the physical and social environment, and this process requires taking constant action in the world while keeping our bodies moving. Unlike mechanical systems like cars that can be stopped to be “repaired”, living systems cannot stop moving because a lack of bodily movements (e.g., cardiac signals or breathing) will entail death. However, because living systems such as human bodies are finite systems resisting entropy (Damasio, 2000; Friston, 2005; Ciaunica, 2025), it is crucial to strike a balance between what sensory information to ignore and what sensory information to prioritize on the fly, hence the vital role of the phenomenon of somatosensory attenuation linked to dynamic embodied selfhood, which is designed precisely to deal with this fine balance between attending and dis-attending self- and world-related information in time. To put it provocatively, too much information kills the information because time and energy are limited in living systems (Ciaunica, 2025). This means that the finite range of energetic resources we have access to forces living systems to process information qualitatively rather than quantitatively.

Effective coupling between bodily movements and bodily actions relies crucially on the temporal coherence of rhythmic signals from both inside and outside the body. For example, cardiac signals (e.g., cardiac cycle and phase) and walking rhythms (e.g., gait cycle and phase) need to phase lock to provide a coherent sense of the bodily self in time. We suggest that somatosensory attenuation allows healthy individuals to dynamically prioritize contextually relevant external sensory inputs over self-related signals, which are treated as less salient due to their regularity. As we discussed above, bodily self-awareness emerges from the dynamic multisensory integration of interoceptive signals (e.g., heartbeat), proprioceptive feedback (e.g., from walking), and exteroceptive cues (e.g., the sound of one’s own footsteps). This integration aligns inner and outer rhythms, allowing the body to feel “anchored” in the world. Additionally, coupling in bodily rhythms requires integration across multiple temporal scales, including real-time observations (e.g., heartbeat and step timing), intermediate dynamics (e.g., phase locking in seconds), and higher-level beliefs (e.g., self-coherence over minutes, hours, and years).

Here, we used the PP framework to suggest that in DP, amplified sensory precision and persistent prediction errors disrupt this multi-timescale integration. This results in short-term desynchronization, where, for example, cardiac and walking signals become misaligned, and long-term fragmentation, impairing the coherence of the self model and contributing to the feelings of detachment and non-existence characteristic of DP. This sense of alienation is cemented by a lack of trust in a self model at a higher metacognitive level. Although individuals with DP “know” they are the agents of their actions, it does not feel like it. This misalignment between the cognitive and experiential levels exacerbates the sense of disconnection: “It feels like something else is controlling my hands; like I’m a puppet, and the words aren’t anything I’m playing an active role in dreaming up. It’s weird how diametrically opposed my feelings and logic can be”—Perkins (2021).

Indeed, in DP, heightened precision of self-related sensory signals may generate ascending prediction errors. These errors arise because it is impossible to predict signals with both overly high precision and minimal errors, especially for a rigid and dysfunctional generative self model. As a result, signals that are normally “transparent” and predictable, such as coupled heartbeats and walking rhythms, appear noisy and unstable. This lack of predictability due to persistent mismatch prevents the model from recalibrating and synchronizing rhythmical self-related sensory perception. The key idea is that the amplified precision of sensory signals in DP thus creates “noise” due to ascending errors or variability in possible signal timing. For instance, over-weighted interoceptive precision may exaggerate small variations in cardiac rhythm, disrupting the generative model’s ability to predict the next cardiac phases accurately. Walking rhythms, in turn, may lose their regularity due to amplified proprioceptive or motor signals or exteroceptive sound, further destabilizing temporal alignment. As a result, without temporal coherence, interoceptive and exteroceptive signals remain uncoupled, fragmenting the sense of bodily coherence.

The core argumentative streamline can be summarized as follows:

The sense of self depends on multisensory integration of interoceptive, exteroceptive and proprioceptive bodily signals. Living systems such as human bodies move constantly, paced by regular sensorimotor rhythms. Highly predictable sensorimotor rhythmic information is accompanied by sensory (e.g., proprioceptive) attenuation which is a key mechanism for a healthy sense of self. Because the world is a constant source of both predictable and unpredictable information, a healthy sense of self depends on the fine-tuned regulation of the link between attending and dis-attending self-related bodily signals. The Predictive Processing view offers a compelling theoretical and computational framework explaining the mechanisms of sensory attention and sensory attenuation. Here, we suggested that when such attenuation fails in DP, self-caused movements are experienced no differently from other-caused ones, leading to a phenomenology of detachment from the body^1^.

If our hypotheses are correct, then one straightforward prediction is that self-related signals in DP, including interoceptive signals (e.g., heartbeat) and exteroceptive signals (e.g., the sound of footsteps while walking), are amplified rather than attenuated as they should be in adaptive systems. Adopting a Bayesian Active Inference perspective on the disrupted self in DP—one that emphasizes rhythmic coupling between internal bodily cycles and outward movement—opens several avenues for both basic research and clinical intervention. First, viewing the sense of self as an ongoing process of dynamic synchronization encourages novel experimental paradigms that jointly measure inner movements like cardiac or respiratory, and actions like locomotor rhythms, alongside subjective reports of bodily self-consciousness. Such paradigms could shed light on how coupling mechanisms evolve over time and how they may be disrupted in disorders like DP.

A second prediction is that training individuals with DP to synchronize their bodily movements and bodily actions may result in lowering their alienation symptoms, making them feel more connected with their self and body. We also expect to find synchronization between cardiac and locomotor signals in typical but not in DP individuals. For example, there is a robust body of work outlining the key role of cardio-locomotor synchronization in optimizing blood circulation and hence performance (Kirby et al., 1989; Niizeki et al., 1996; Niizeki, 2005; Novak et al., 2007; Rouffet et al., 2008; Niizeki & Saitoh, 2014; Takeuchi et al., 2014; Wittstein, 2016; De Bartolo et al., 2021; Wakeham et al., 2023). We suggest that examining the dynamic interplay between bodily movements (i.e., cardiac signals) and bodily actions (i.e., footsteps) in people with low and high occurrences of DP experiences may help us better understand the disrupted mechanisms underlying DP experiences. Specifically, if altering the sounds produced by one’s bodily actions changes body perceptions and has simultaneous effects on changes inside the body (e.g., heartbeats), then these modulations may enhance the sense of self and sense of presence in the world in people experiencing DP. This is because dynamic sensory feedback may increase not only (a) the feeling of being in control of one’s body and actions but also (b) the malleability of one’s sense of self, allowing people to feel less ‘stuck’ in one’s head (mind) and ‘putting them back’ into their bodies.

Moreover, therapeutic approaches might benefit from deliberately restoring or enhancing rhythmic coherence—for instance, through structured movement practices (e.g., dance or gait training) with biofeedback-based interventions that help to synchronize with cardiac and respiratory signals. However, it is crucial that these interventions remain implicit rather than explicit, as implicit practices can support the adaptive attenuation of self-related signals and prevent counterproductive over-attention to them. This aligns with evidence suggesting that active, embodied engagement recalibrates attention away from hyper-reflexive self-monitoring, fostering a more fluid integration of internal and external cues (Ciaunica et al., 2022; Millman et al., 2023).

Finally, and speculatively, we suggest that the common distinction between signals situated inside and outside the body is potentially misleading in this new framework we suggest here. For example, treating heartbeats as purely interoceptive signals and visual stimuli (like seeing one’s own reflection in a mirror) as exclusively exteroceptive tacitly presupposes a clear-cut distinction between the inner and outer body. Yet, information about heartbeats can be perceived both interoceptively (through internal bodily sensations) and exteroceptively (e.g., hearing our own heartbeat or visually observing it on a live cardiogram or feeling the pounding chest). Similarly, lung activity (i.e., signals originating inside the body) can also be felt and externally observed through the rise and fall of our chest. This process is necessarily accompanied by the sound of our breath, creating a multisensory synchrony between internal sensations, external visual feedback, and auditory cues. Such synchronization of bodily signals reinforces the coherence of self-perception, anchoring our sense of ownership and agency over the body. At any point, there cannot be a gulf between signals coming from inside versus outside the body. In living systems, there is only one single sensory system: the moving body.

One open question is how exactly (i) altered somatosensory attenuation and (ii) altered multisensory integration are related to each other, thereby leading to the pathophysiology in DP. Which one of these mechanisms comes first and entrains the other in shaping the predictive model of altered selfhood in DP? Indeed, it is important to establish whether diminished sensory attenuation (diminished downregulation of PE gain) or upregulation of prediction error gain is fundamental to DP pathophysiology 2. Further work needs to test these two hypotheses by developing computational models in relation to DP. For the purpose of this paper, we remain agnostic on which mechanism is more basic and how exactly they are related. Speculatively however, we suggest that somatosensory attenuation and multisensory integration of self-related information may be two complementary mechanisms acting in tandem. Living bodies are constantly moving systems and are constantly integrating multisensory self- and world-related sensory information. There can be no somatosensory feedback without sensory input and no sensory input without somatosensory feedback. This is because the world is present before the body is present and acts as a constant background against which the body negotiates its own existence. The two mechanisms may be differentiated conceptually, but not biologically, as they inform each other in tandem. Further work is needed to provide a detailed Predictive Processing computational model of this idea and its simulations.

## 7. Conclusions

This paper outlined the importance of embodied and active engagement with the world in building a coherent sense of self within a volatile environment. We argued that one overlooked yet crucial aspect of this picture is that our sense of self depends on adaptively coupling bodily movements and bodily actions. We saw that a promising theoretical framework to address this complex question is provided by the influential Predictive Processing (PP) and Active Inference frameworks. We highlighted the key role of striking the balance between sensory attending and sensory dis-attending or attenuating self-related information as a key component of embodied selfhood in healthy individuals. The pervasive background of our experiences is not only the embodied self but the moving embodied self. Specifically, we suggested that precisely because our inner bodily self is inherently moving and rhythmical, these rhythms are central to our embodied sense of self and active presence in the world (Park & Tallon-Baudry, 2014; Corcoran et al., 2023/2025). Crucially, these are also the processes we need to attenuate the most in order to ensure smooth engagement with the world. Paradoxically, we perceive the world as a continuous flow precisely because its fluidity is punctuated by rhythms, rollercoasting the ups and downs of sensory signals into a dynamic harmonious stream. When this coupling is disrupted, the world and self appear fragmented, as in the case of Depersonalization Disorder, a condition that makes people feel detached from the self and body. If our hypotheses are correct, this means that individuals who move more in the world are also more successful in integrating multisensory self-related information and have a healthier sense of self. Paradoxically, the more one is actively connected and engaged with the world, the more one is connected with one’s self. This hypothesis may have a profound impact on potential therapy for self-disturbances in various conditions such as depersonalization, psychosis, and schizophrenia, by focusing on repairing the dynamical bridge between the world and self, rather than the self alone.

## Data Availability

Not applicable.

## Notes

1 We would like to thank the Reviewer for pressing clarification on this point and providing useful feedback on how to better phrase the argument.

2 We would like to thank one Anonymous Reviewer for pressing clarification on this key point.
